# Low to near-zero CO_2_ production of hydrogen from fossil fuels: critical role of microwave-initiated catalysis

**DOI:** 10.1098/rsta.2024.0061

**Published:** 2025-05-22

**Authors:** Xiangyu Jie, Daniel R. Slocombe, Adrian Porch, Tiancun Xiao, Sergio González-Cortés, Saud Aldrees, Jon R. Dilworth, Benzhen Yao, Martin-Owen Jones, Vladimir Kuznetsov, Peter P. Edwards

**Affiliations:** ^1^ Department of Chemistry, Queen Mary University of London, London, UK; ^2^ School of Engineering, Cardiff University, Cardiff, South Glamorgan, UK; ^3^ Department of Chemistry, University of Oxford, Oxford, UK; ^4^ King Abdulaziz City for Science And Technology, Riyadh, Riyadh Province, Saudi Arabia; ^5^ ISIS Facility, Rutherford Appleton Laboratory, Oxford, UK

**Keywords:** microwave-initiated catalytic pyrolysis, hydrogen, catalysis

## Abstract

Presently, there is no single, clear route for the near-term production of the huge volumes of CO_2_-free hydrogen necessary for the global transition to any type of hydrogen economy. All conventional routes to produce hydrogen from hydrocarbon fossil fuels (notably natural gas) involve the production—and hence the emission—of CO_2_, most notably in the steam methane reforming (SMR) process. Our recent studies have highlighted another route; namely, the critical role played by the microwave-initiated catalytic pyrolysis, decomposition or deconstruction of fossil hydrocarbon fuels to produce hydrogen with low to near-zero CO_2_ emissions together with high-value solid nanoscale carbonaceous materials. These innovations have been applied, firstly to wax, then methane, crude oil, diesel, then biomass and most recently Saudi Arabian light crude oil, as well as plastics waste. Microwave catalysis has therefore now emerged as a highly effective route for the rapid and effective production of hydrogen and high-value carbon nanomaterials co-products, in many cases accompanied by low to near-zero CO_2_ emissions. Underpinning all of these advances has been the important concept from solid state physics of the so-called Size-Induced-Metal-Insulator Transition (SIMIT) in mesoscale or mesoscopic particles of catalysts. The mesoscale refers to a range of physical scale in-between the micro- and the macro-scale of matter (Huang W, Li J and Edwards PP, 2018, Mesoscience: exploring the common principle at mesoscale, *Natl. Sci. Rev*. **5**, 321-326 (doi:10.1093/nsr/nwx083)). We highlight here that the actual physical size of the mesoscopic catalyst particles, located close to the SIMIT, is the primary cause of their enhanced microwave absorption and rapid heating of particles to initiate the catalytic—and highly selective—breaking of carbon–hydrogen bonds in fossil hydrocarbons and plastics to produce clean hydrogen and nanoscale carbonaceous materials. Importantly, also, since the surrounding ‘bath’ of hydrocarbons is cooler than the microwave-heated catalytic particles themselves, the produced neutral hydrogen molecule can quickly diffuse from the active sites. This important feature of microwave heating thereby minimizes undesirable side reactions, a common feature of conventional thermal heating in heterogeneous catalysis. The low to near-zero CO_2_ production of hydrogen via microwave-initiated decomposition or cracking of abundant hydrocarbon fossil fuels may be an interim, viable alternative to the conventional, widely-used SMR, that a highly efficient process, but unfortunately associated with the emission of vast quantities of CO_2_. Microwave-initiated catalytic decomposition also opens up the intriguing possibility of using distributed methane in the current natural gas structure to produce hydrogen and high-value solid carbon at either central or distributed sites. That approach will lessen many of the safety and environmental concerns associated with transporting hydrogen using the existing natural gas infrastructure. When completely optimized, microwave-initiated catalytic decomposition of methane (and indeed all hydrocarbon sources) will produce no aerial carbon (CO_2_), and only solid carbon as a co-product. Furthermore, reaction conditions can surely be optimized to target the production of high-quality synthetic graphite as the major carbon-product; that material of considerable importance as the anode material for lithium-ion batteries. Even without aiming for such products derived from the solid carbon co-product, it is of course far easier to capture solid carbon rather than capturing gaseous CO_2_ at either the central or distributed sites. Through microwave-initiated catalytic pyrolysis, this decarbonization of fossil fuels can now become the potent source of sustainable hydrogen and high-value carbon nanomaterials.

This article is part of the discussion meeting issue ‘Microwave science in sustainability’.

## 1. Introduction

Hydrogen has again emerged as a potential option for global decarbonization [1–5]. Unfortunately, as noted in a recent contribution by Baroness Brown of Cambridge [6] ‘…*hydrogen has been hyped as the silver bullet that can decarbonize almost everything’*. In addition, as well as various safety and environmental aspects [7], significant scientific, technical and economic barriers remain along the pathway to its acceptance as a major energy source. Perhaps the most pressing issue is the need—recognized decades ago—for a low-, ideally zero-carbon-emissions route for its mass production [8,9]. The current large-scale industrial production of hydrogen uses fossil fuels and water through the process of the steam reforming of methane (SMR).

SMR consists of several different catalysed reactions, but it is typically described by reaction equation (1.1):


(1.1) $$CH_{4}+2H_{2}O\to CO_{2}+4H_{2}$$

Although SMR is universally recognized as a highly efficient, long-standing process, of course, it leads to the emission of large quantities of CO_2_ into the atmosphere [8–10].

In an effort to render this a CO_2_-free production route for hydrogen, it is being actively discussed that this CO_2_ must be captured and sequestered under the ocean or underground through the process of Carbon Capture and Storage (CCS). This is an energy-intensive and costly process [11,12] and—inevitably—must surely be associated with ecological uncertainties, not to mention widespread societal concerns relating to the sequestration of huge quantities of CO_2_ [13].

One may surely ask why should we produce tens of millions of tons of CO_2_ (‘aerial carbon’) and then spend yet-further hundreds of millions of pounds in energy-intensive processes to capture, transport and dispose of it at potentially great significant risk, when the production of CO_2_ should be avoided in the first place?

We highlight here an entirely different option, first advanced several decades ago; a fundamentally different tactic for producing hydrogen from fossil fuels, but now with low or zero CO_2_ emissions [14–18]. To appreciate this approach, we note that it is the use of water (steam) in the reaction with the fossil fuel hydrocarbons (as typified in the SMR process, equation (1.1)) which leads to CO_2_ emissions. Thus, in the present worldwide processes employed for the generation of hydrogen from fossil hydrocarbons, all the constituent carbon in the hydrocarbons ends up as the oxides of carbon, either CO or mostly CO_2_!

The alternative approach presented here requires that the source of hydrogen *must only be the hydrocarbon itself* which can undergo thermal decomposition or pyrolysis, or cracking reaction via:

(1.2) $$CnHm\;-\to nC+m/2H_{2}.$$

Thus, in the case of methane:


(1.3) $$CH_{4}\to C+2H_{2}.$$

**Figure 1.**
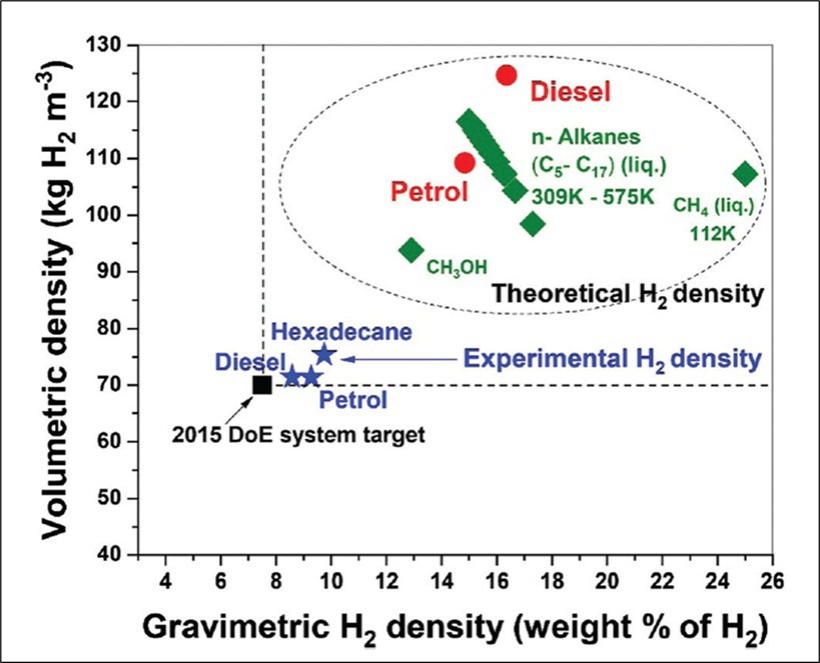
The intrinsic gravimetric and volumetric densities of hydrocarbon fossil fuels as sources of hydrogen. While the experimental results surpass the US Department of Energy’s 'Performance/System Targets' for hydrogen storage materials, presently, they achieve only about half of the theoretical gravimetric and volumetric densities of these hydrocarbons. In other words, higher hydrogen densities can be achieved by improving selectivity and efficiency [19].

This alternative platform [14–18] takes advantage of the very high, intrinsic hydrogen content of methane—and indeed, many other fossil hydrocarbons (figure 1)—but in such a way—ideally—to release only their constituent hydrogen but with no attendant CO_2_ production. Interestingly, both the intrinsic gravimetric and volumetric densities of these hydrocarbon fossil fuels as hydrogen sources exceed the US Department of Energy ‘Performance Targets’ for hydrogen storage materials.

The thermal decomposition of the simplest and most abundant hydrocarbon, methane—and the one with the highest gravimetric hydrogen density—to yield hydrogen and solid carbon is indeed a well-known process, long applied to the industrial production of soot. However, the process requires very high temperatures, *ca*. 900°C and above. This derives from the strong C–H chemical bond in methane (440 kJmol^−1^). Obviously, the realization of the thermal decomposition or deconstruction of the C–H bonds in methane and other hydrocarbon fossil fuels at, ideally, lower temperature, requires the use of catalysts [8–10].

In such a heterogeneous catalytic process, for example, the conventional heating of a metal catalyst particle is invariably a slow and energy-consuming process, arising from the obvious condition that the hydrocarbon component—inevitably in vast excess—is, of course, a low thermal-conductivity medium. Thus, bringing the individual metal catalyst particles to the necessary reaction temperature to activate any chemical C–H bond-breaking necessitates the propagation of thermal energy, initially from the external container wall, through the interior of the hydrocarbon medium, ultimately leading to the required localized heating of the individual, dispersed metal catalyst particles [20].

By contrast, microwave heating in such a heterogeneous catalytic reaction will be driven by the selective absorption and conversion of incident microwave electromagnetic energy *directly to the metal catalyst particle itself*. The incoming microwave energy is then effectively transferred only to the metal catalyst particles with the hydrocarbon ‘host’—to all intents and purposes—being transparent to microwaves. Such a microwave-initiated heterogeneous catalytic process therefore negates the requirement of an inefficient, conventional heat transfer process to the catalyst to effect a chemical catalytic reaction [20].

Our studies over the recent past have indeed revealed the remarkable ability of incident electromagnetic radiation in the form of microwaves to effect a rapid heating of supported and free metal catalyst particles, particularly in the so-called ‘Mesoscopic size regime’, to promote both rapid and highly effective catalytic C–H bond scission in both gaseous (CH_4_) liquid and solid hydrocarbons and biomass [19–24]. This list also includes natural hydrocarbon fossil fuels such as petroleum and even crude oil—to yield ultra-low or, in some cases, CO_2_-free hydrogen—and solid carbon nanomaterials. We review here these advances in microwave-initiated catalysis, both in a wide range of fossil hydrocarbon fuels and indeed in plastics waste also.

**Figure 2.**
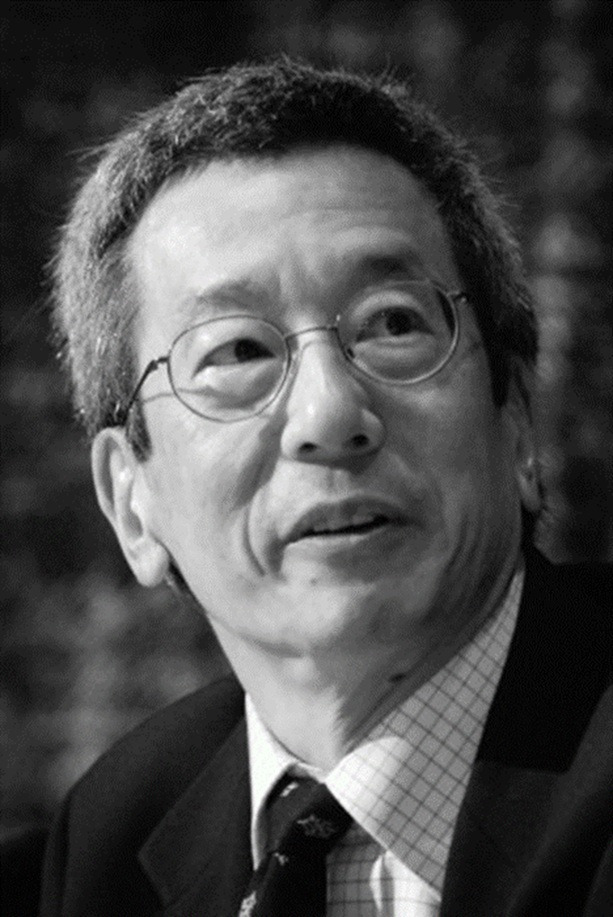
Professor Roger Tsien, ForMemRS, Chemistry Nobel Laureate. (Credit: Holger Motzkau/Wikipedia/Wikimedia Commons.)

When visiting Oxford to present the 2015 Alan Katritzky Lecture, Nobel Laureate Professor Roger Tsien, ForMemRS, (figure 2), was shown the instantaneous and large-scale evolution of H_2_ from the fossil hydrocarbon wax, under microwave-initiated catalysis conditions in our laboratory fume hood. Professor Tsien exclaimed: *‘Remarkable, Peter, and Colleagues: This must surely be a revolution for the rapid production of hydrogen!’*

As we will hope to illustrate, at the heart of these innovations are the completely interlinked issues not only of the size-dependence of the catalytic, but also of the electronic-properties, of small, nominally metallic particles.

## 2. On size-dependent catalysis

The interesting variation of catalytic activity of metals with their physical size below a certain threshold (say, 10 nm) has previously been attributed to most of—if not all—the intrinsic physicochemical properties of metals that show size-dependence. As indeed noted in important work by G. C. Bond (private communication to Peter Edwards, 2010), the first and, perhaps, most obvious effect is the inevitable rising fraction of surface atoms as the catalyst particle size diminishes, especially for those atoms having very low coordination numbers. Of course, as one moves into the so-called mesoscopic size regime, for example, in the size range 10−50 nm, an appreciable percentage of atoms, some 10% and above, are now found at the surface of the metal particle.

However, whatever the scale and extent of such ‘microstructural’ factors in any size-dependent catalytic process, understanding the inevitable changes in the basic, fundamental electronic constitution of individual, very small particles cannot be overstated [25,26]. Importantly, it was stressed a long time ago [27,28] that the physical surface of a small ‘metallic’ particle serves not only to scatter the included or embedded conduction electrons, but also determines the very quantum eigenstates of the particle. Here, wavefunctions are now necessarily bound within a finite volume, rather than simply being free (i.e. itinerant) electronic states scattered by the particle’s surface. Indeed, there are strong arguments that this aspect may indeed be *the* dominant feature in dictating the fundamental nature, and performance, of catalyst particles in the mesoscopic size regime; something that we certainly believe to be the case in the microwave-initiated heating and catalytic properties of mesoscopic metal particles.

## 3. The Metal Divided: the Size-Induced Metal–Insulator Transition

There are countless examples in nature where highly conducting metals or metallic materials are transformed into stubbornly resistive insulators or non-metals by changes in pressure, temperature or chemical composition; this is the Metal-to-Insulator Transition, the MIT [29–38]. To this list we add the intriguing concept and possibility of a Size-Induced Metal-to-Insulator Transition (SIMIT); namely, a transition occurring entirely within a single, isolated small particle or cluster of a bulk metal [39–43]. Such an electronic and thermodynamic transition, from a highly conducting metal particle to a non-conducting (or feebly conducting) ‘metal’ particle, is a safe prediction for any particle or cluster of atoms of a chemical element of the Periodic Table that is a metal in bulk form and now subject to division in its physical size [44]. There is now considerable, accumulated evidence (some of it outlined below) to show that below a certain physical size—invariably in the mesoscopic size regime—individual particles of nominally ‘metallic’ elements now become non-metallic *and* non-conducting.

**Figure 3.**
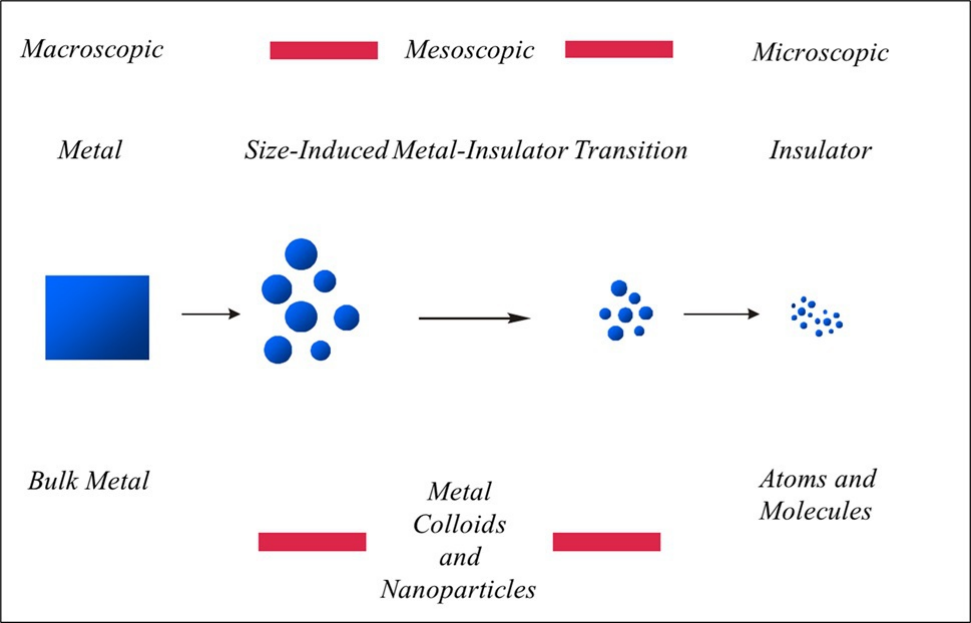
The Metal Divided: a representation of the successive fragmentation or division of a single grain of metal [41].

A schematic representation of the effects of the successive division or fragmentation of a single grain of a ‘nominally’ metallic bulk catalyst particle is highlighted in figure 3; this attempts to set out many of the global features associated with the successive fragmentation of a piece of metal [39,41–43].

Interestingly, perhaps the earliest discussion of this effect dates back to 1857 in the prescient work of Faraday on colloidal gold, silver and other metals which led him to conclude that… *‘…the gold is reduced in exceedingly fine particles which, becoming diffused, produce a beautiful fluid…the various preparations of gold, whether ruby, green, violet or blue…consist of that substance in a metallic, divided state’* [45].

Particularly relevant for our present purposes is that the critical point or region for the SIMIT transition appears to be close to, or within, the size range of many operational, heterogeneous metal catalyst particles.

To understand the origins of the SIMIT, one must understand the fundamental electronic properties of typical catalytic metal particles ranging from a few tens, to a few hundreds of nanometres, in size [25–28,39–43,46–49]. Within this size regime, mesoscopic catalyst particles are therefore intermediate in physical size between the microscopic regime (nm) on the one hand, and the macroscopic bulk state of matter on the other [41,43]. Such particles in this characteristic size regime challenge conventional thinking, since—remarkably—it is their characteristic physical size which now governs both their fundamental physical and chemical properties. Thus, this size-dependent electronic structure determines the fundamental metallic or insulating status of an individual particle. By varying *only* the physical size of an individual mesoscopic catalyst particle, its electronic properties can be adjusted between the full extremes of a metal and that of an insulator. As we will illustrate, this includes their unique and important catalytic properties under the influence of incident microwave electromagnetic radiation.

To understand the changes in electronic structure and behaviour upon this subdivision of a piece of metal, we note that metallic properties derive from the existence of a partially occupied band with an electronic energy level spacing infinitesimally small near the Fermi level, *E_F_*, so that a small external potential can create electron–hole pairs, allowing a flow of electric current. Importantly, above *E_F_* there also exists an infinite number (i.e. a continuum) of infinitesimally separated (and empty) electronic energy levels, which will be populated for electronic conduction under the application of an applied electric field [41].

**Figure 4.**
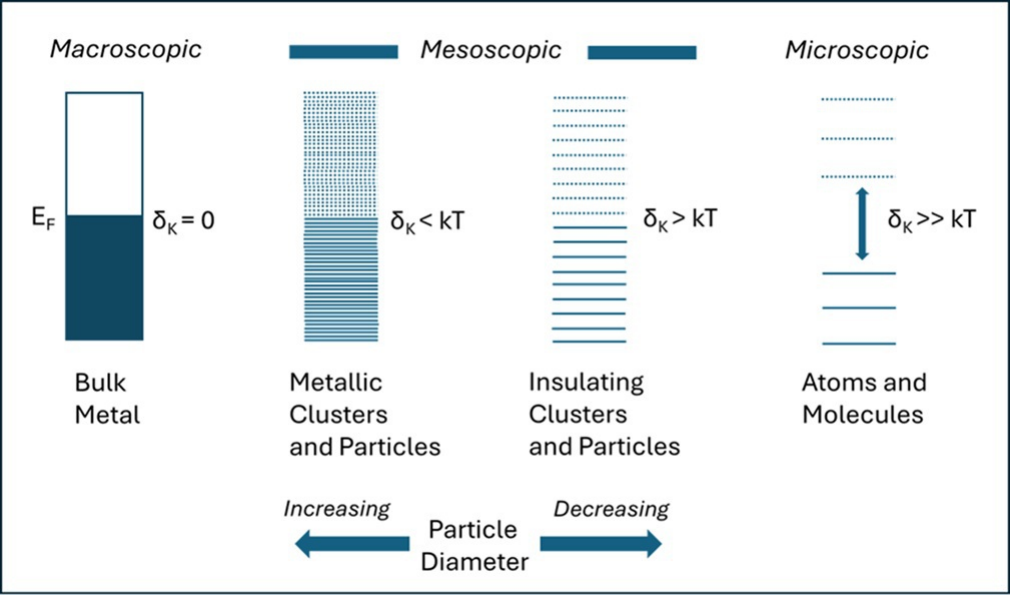
The Size-Induced Metal-Insulator Transition: the evolution of the Kubo electronic energy gap as the physical size of a metal particle is continuously reduced from the *Macroscopic* through the *Mesoscopic* to the *Microscopic* regimes. In all cases, we identify the approximate particle size regime and the accompanying (Kubo) average electronic energy gap separating occupied and unoccupied electronic energy levels as compared with thermal energy (kT)mat the observation temperature [41].

Kubo [28] pointed out some time ago that when a metal particle is sufficiently finely divided down to mesoscopic or microscopic dimensions, that characteristic continuum of electronic energy levels that we associate with a metal, will now give way to a manifold of discrete energy levels, with an average energy separation, δ_K_, between levels of some E_F_/N, where N is the number of atoms contained within a single metal particle. Critically, it is generally proposed that when δ_K_, the so-called Kubo energy gap, becomes comparable with the (ambient) thermal energy, kT, the electronic energy levels of the particle now become discrete, rather than continuous. However, the occurrence of any transition from a continuous—to a discrete—manifold of energy levels (i.e. for any case for which δ_K_ is finite) will signal a major and fundamental change in electronic properties of the individual particle. A schematic representation of the size-dependent electronic structure of (nominally) metallic particles, the emergence of the Kubo gap and the SIMIT is given in figure 4.

It is important to note that such a metal–insulator transition in a finite system, such as an individual catalyst particle, is not a discontinuous or first-order electronic phase transition like those found in many macroscopic systems; e.g. doped semiconductors, or some transition metal oxides. Rather it is a continuous transition which occurs gradually with a decreasing particle size [41].

**Figure 5.**
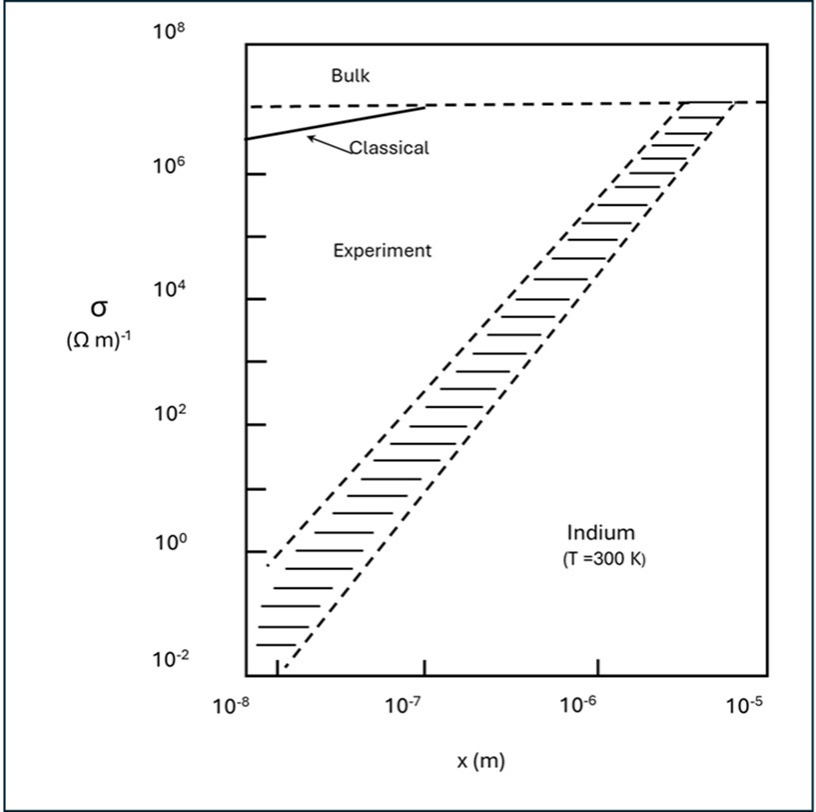
The Metal Divided: the size-dependent quasi-DC conductivity of mesoscopic indium particles versus the particle sizes. Note the log–log plot. For comparison, the bulk conductivity and the classical size effect are displayed to highlight that this massive drop in conductivity in diminishing particle sizes cannot be accounted for in terms of a classical (i.e. non-quantum) effect. (Reproduced from [40], with permission from Elsevier.)

Clear experimental work for a SIMIT in mesoscopic, nominally ‘metallic’ conductors has been found in the pioneering studies of Marquardt, Nimtz and co-workers [40,46–48]. In figure 5, we show the measured electrical conductivities (within each particle) versus particle size for In particles of different sizes. Those workers also find similar examples of the SIMIT in an impressive variety of metals, notably the ‘metallic’ elements Ag, Co and Pt from the Periodic Table.

Note that the clear evidence for a continuous SIMIT as the conductivity within each crystal or particle drops by more than eight orders of magnitude as the particle size is reduced. Importantly, those authors note that any classical interpretation of the SIMIT fails completely. They stress that the SIMIT is the result of a quantum behaviour of the electronic structure within the individual mesoscopic particle and can only be interpreted on the basis of the wave nature of electrons [49]. On moving to larger and larger particle sizes, those authors also note that discreteness of the electronic energy level structure persists up to submicron particle sizes!

We took this as sound theoretical and experimental evidence that the practical catalyst particles employed in our studies, having dimensions close to, or below, the SIMIT, would exhibit ‘non-metal’, i.e. non-conducting, electronic behaviour as compared with their bulk, highly conducting parent ‘metals’.

We believed that, in stark contrast to the situation for a bulk metal, such mesoscopic catalyst particles would, therefore, have the inherent ability to absorb microwaves and heat most effectively when placed in a microwave electric field.

To further explore this possibility theoretically, Porch *et al.* [50] calculated the total electromagnetic absorption in non-magnetic small conducting particles within microwave fields as a function of conductivity and mean particle radius for a range of particle sizes and conductivities. They concluded that the magnetic absorption dominates electrical absorption over a wide range of radii for highly conducting particles with an optimum absorption set by the ratio of the mean particle radius to its microwave skin depth. Importantly, this indicates that for metal particles of any conductivity, optimized magnetic absorption and hence the microwave heating of said particles by magnetic induction can be achieved by the simple selection of the mean particle size.

**Figure 6.**
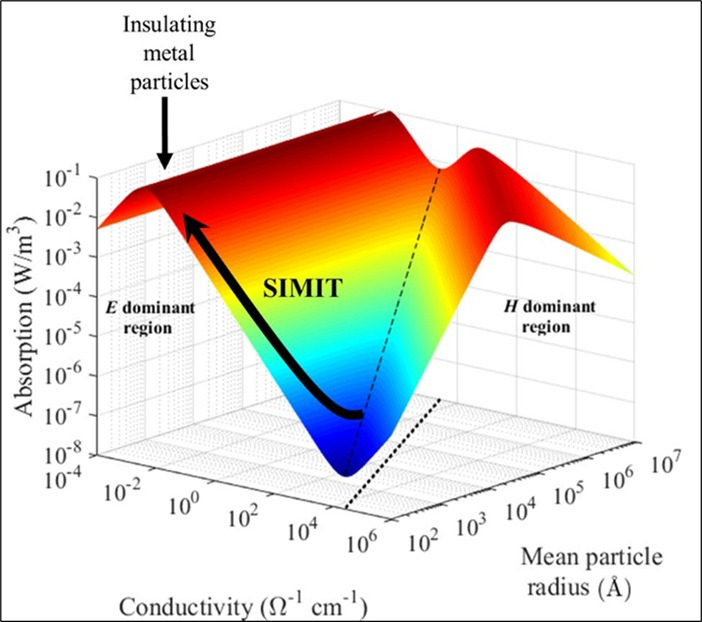
The total electromagnetic absorption across the Size-Induced Metal-Insulator Transition (SIMIT) in isolated non-magnetic particles as a function of conductivity and mean particle radius. The optimal electric absorption and optical magnetic absorption are marked in red. As the particle size is continually reduced across the SIMIT, we see the transition to ‘insulating metal particles’ [50].

We note that while microwave photons (approx. 10^–5^ eV at 2.45 GHz) lack sufficient energy to *directly* excite electronic transitions (typically requiring eV-level energies), mesoscopic catalyst particles near the SIMIT exhibit enhanced microwave absorption due to their unique electronic structure. As conductivity within each particle decreases in this regime (figure 5), particles transition from reflecting to absorbing microwaves via collective electromagnetic interactions, such as magnetic induction and eddy current losses. This absorption generates localized Joule heating, enabling catalytic activation. This mechanism, detailed by Porch *et al*. [50], aligns with the optimal absorption observed when particle size matches the microwave skin depth (figure 6).

Thus, as a catalyst particle size is gradually reduced to, and beyond, a critical size one expects a huge increase in microwave absorption (over some eight orders of magnitude) at the same time as the huge decrease in electrical conductivity within the particle. Paradoxically, this marks the occurrence of what they term ‘insulating metal particles’ (as highlighted in figure 6). Such particles should now exhibit high microwave absorption through collective electromagnetic interactions, including magnetic induction and eddy current losses. This absorption induces localized Joule heating, facilitating catalytic activation, as we shall show in the following sections, highly effective microwave heating and catalytic behaviour for a range of applications, particularly in the catalytic decomposition of hydrocarbons to yield hydrogen with minimal or zero CO_2_ emissions.

## 4. Size-dependent microwave heating and catalytic activity of fine iron particles: deep dehydrogenation of a model hydrocarbon, hexadecane

**Figure 7.**
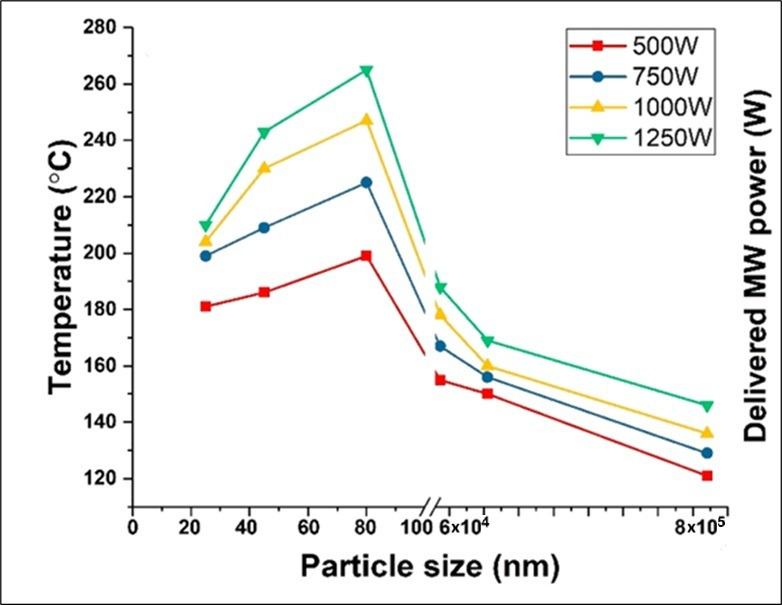
A study of the effect on Fe particles of various sizes exposed to microwave irradiation and the resulting temperature (as measured by a pyrometer) at 600 s for various incident microwave powers [24].

In a comprehensive study, Jie *et al.* investigated the interaction between microwave radiation and Fe particles of different sizes in the catalytic deep dehydrogenation of a model hydrocarbon, hexadecane [24]. They found that the size-dependent electronic transition of Fe particles from a microwave ‘reflector’ to a microwave ‘absorber’ produced highly efficient heating of the individual microwave absorbing particles and, with that, corresponding highly effective catalytic properties in the resulting dehydrogenation reaction. They first measured the microwave-initiated temperature rise and showed that this was strongly size-dependent among Fe catalyst particles of different sizes. The temperature rise (for a given incident microwave power) achieved in various particle sizes was in the order 80−60 nm > 45–30 nm > 25 nm > 74 microns > 210 microns > 841 microns. The temperature profiles of Fe particles of various sizes under microwave irradiation with various input powers are shown in figure 7.

**Figure 8.**
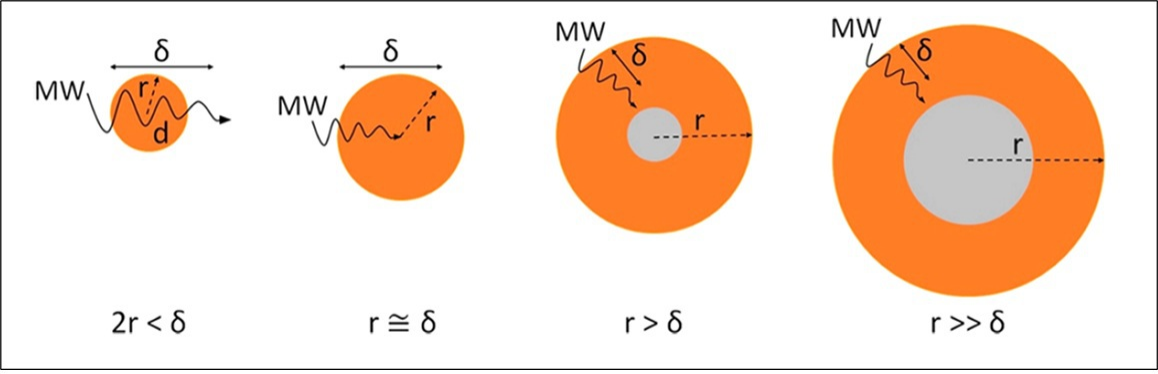
A schematic representation of the microwave penetration in different sized Fe particles having the same skin depth at a frequency of 2.45 GHz. Here, r and δ are the particle radius and skin depth of the particles, respectively [24].

At the operating microwave frequency of 2.45 GHz, different particles of Fe have the same skin depth, approximately 41.5 nm (MUST online calculators for electronics. http://mustcalculate.com/electronics/skindepth.php; accessed 18-03-2025). Therefore, the actual volume of the Fe catalyst particle which absorbs incoming microwave power is very closely linked to the particle size and the skin depth. For example, when the particle size decreases below its skin depth—from 41.5 nm to 30 nm—less energy is absorbed due to the smaller iron volume compared with Fe particles with a size of 80 nm. As a result, the temperature achieved is lower, and the heating rate is reduced. A schematic representation of the situation for different sized Fe particles is shown in figure 8.

We also note that microwave frequency has a direct influence on skin depth. Higher frequencies reduce microwave penetration, confining heating primarily to the particle surfaces, whereas lower frequencies allow for deeper energy absorption. This variation in microwave skin depth therefore plays a crucial role in determining the efficiency and uniformity of heating under microwaves, particularly in catalytic and material processing applications, where controlled energy distribution is essential for optimizing performance.

Obviously, for any catalytic process to occur, the catalyst particles must reach the necessary temperature to initiate any chemical reaction. When the diameter of the Fe particle is smaller than its skin depth at microwave frequencies, the full volume of the Fe particle will absorb the incident microwave radiation and generate heat. By contrast, when the radius of the catalyst particle becomes larger than the skin depth, only a fraction of the particle will absorb the incident microwave radiation, and that only throughout the skin depth (figure 8).

**Figure 9.**
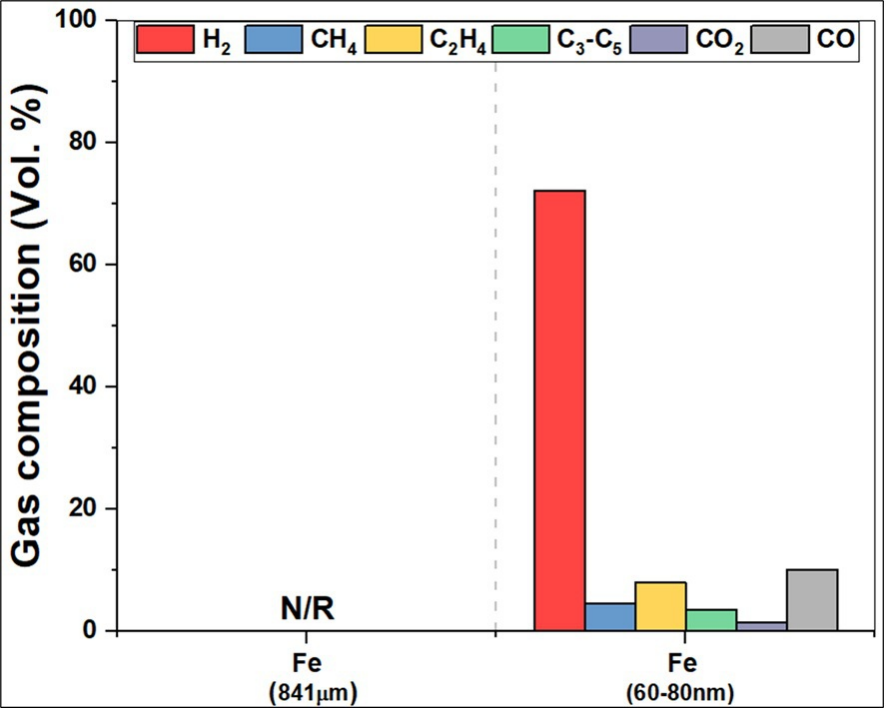
A comparison of the generated gases from two sets of Fe catalysts of different particle sizes in the microwave-initiated dehydrogenation of hexadecane. N/R denotes no catalytic reaction for the larger (841 micron) particles [24].

To highlight how changing the particle size affects the corresponding microwave-initiated catalysis, in figure 9, we compare the catalytic performance in the dehydrogenation process of two sets of free-standing Fe particles; one 841 micro and the other 60−80 nm diameter particles. As is clearly evident, the latter Fe catalyst particles show a highly effective catalytic dehydrogenation of hexadecane under microwave initiation. In stark contrast—and remarkably—no measurable catalytic reaction was observed from the 841 micron Fe particles. This arises from the fact that they were heated poorly under microwave initiation due to their large particle size as compared with the microwave skin depth (figure 8).

That work nicely demonstrated that one can determine an optimal particle size for the most effective microwave-harvesting catalysis for any particular chemical reaction, in this case, the deep dehydrogenation of a hydrocarbon.

Microwave-initiated catalysis also provides an excellent method for producing ultra-low CO_2_—or even CO_2_ free—hydrogen from a range of fossil fuels themselves and indeed plastics waste; two areas that we will now briefly review.

## 5. The decarbonization of fossil fuels: hydrogen production with minimal—or zero—CO_2_ emissions

The microwave-initiated catalytic decomposition route developed by our group represents a process for hydrogen production with minimal or near-zero CO_2_ emissions through ‘hydrogen stripping’ or ‘deep dehydrogenation’ of a range of hydrocarbon fossil fuels.

**Figure 10.**
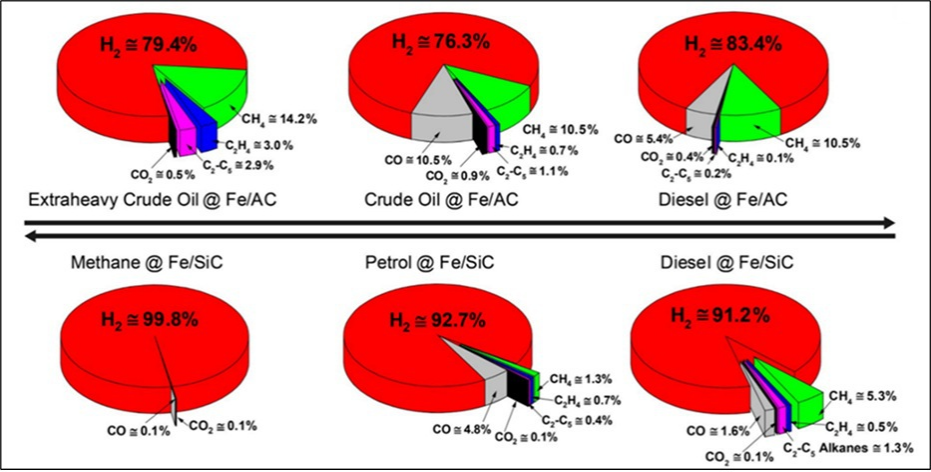
Hydrogen production through the microwave-initiated catalytic dehydrogenation of various fossil fuels. Note, in particular, the very high conversion of methane to hydrogen, exceeding some 99%! [19].

This is tantamount to fossil fuel decarbonization. In addition to using methane as the hydrogen source, we have also discovered that it can readily be applied to heavier natural fossil hydrocarbons—including, even, heavy crude oil! The results of hydrogen stripping from a range of hydrocarbon fuels through microwave-initiated catalysis are shown in figure 10.

At the initiation of the microwave irradiation of the various hydrocarbons, a considerable volume of high purity hydrogen was readily extracted from petroleum and heavy liquid fuel stocks—typically in a period of some 3 min! Defining selectivity as the volume % of the particular product composition of the total gaseous products, we find values of over 90% in the exiting gas stream following the catalytic dehydrogenation of methane, petrol and diesel. For the heavier and obviously more complex crude and extra-heavy crude oil, the selectivity decreased to *ca*. 75–85%. Due to the inevitable extraneous or residual oxygen in certain feedstocks, the catalysts themselves and of course the supports, the production of small amounts of both CO_2_ and CO is understandable.

Turning to methane, we see very high values of conversion, very small concentrations of CO and CO_2_ and—equally important—no other discernible products. We think this is strong evidence that the primary product of this dehydrogenation process on the hot Fe catalyst surface—molecular hydrogen—can rapidly diffuse into the bulk, colder hydrocarbon reaction region around the catalyst with very little time to form other products, as is usually the case with conventional (uniform) heating of the catalytic system. The selective, and highly effective microwave heating of the catalyst particles gives rise to high spatial inhomogeneity and temperature gradients in the reaction medium.

In the microwave-initiated heating, the resulting hydrogen can thus rapidly dissociate from the active sites on the Fe catalyst surface, avoiding many undesirable side reactions.

This clearly also occurs with the liquid hydrocarbons—a phenomenon that has been termed ‘spatial quenching’ of any reaction products.

**Figure 11.**
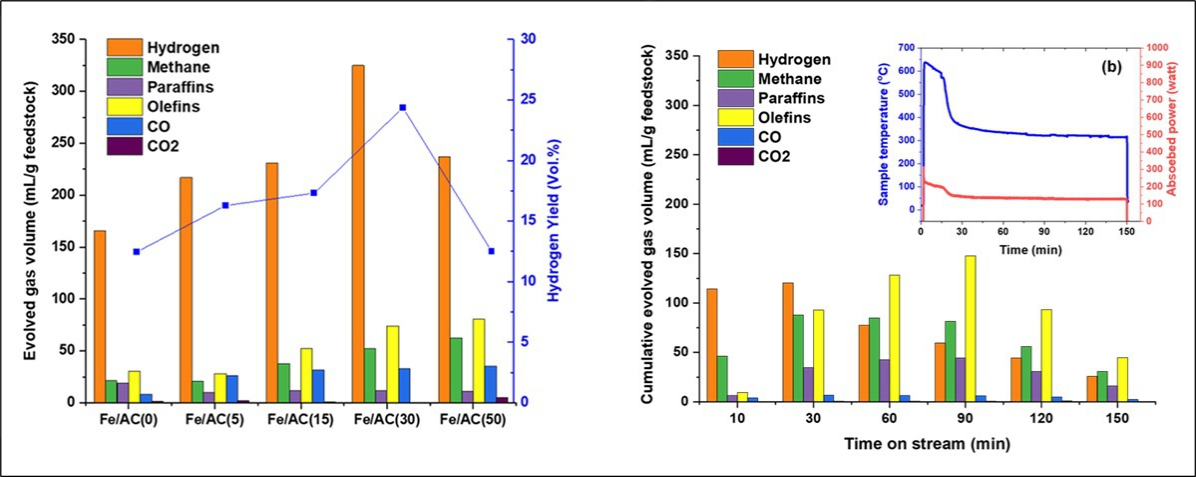
The microwave-initiated catalytic decomposition of Saudi Arabian Light Crude Oil for (*a*) various loadings of Fe catalyst particles on activated carbon and (*b*) the time dependence of the process for a 30 weight% loaded Fe particles on activated carbon [51].

An interesting recent development of the application is the microwave-initiated catalytic process of Saudi Arabian ‘Light Crude Oil’ [51]. Such liquid hydrocarbons themselves possess considerable manipulative convenience and engineering advantages, as compared with their gaseous counterparts. A recent application of microwave-initiated decomposition of hydrocarbons centres on the most widely available natural fossil fuel—that is, Saudi Arabian Light Crude Oil (LCO). Humankind has exploited the large reservoirs of shorter chain ‘light’ oil reserves requiring less energy to extract and refine. This LCO as a liquid petroleum has a low density and flows freely at room temperature. Another attractive feature is that it has low viscosity and a high proportion of light hydrocarbon fractions, thereby giving some of the highest hydrogen content from the hydrocarbon ‘stock’. We have examined a range of Fe catalyst compositions between 0 and 50 wt% using LCO as the hydrocarbon feedstock and activated carbon as a support material. In figure 11, we show the resulting catalytic performances. High levels of catalytic activity for the ‘deep’ dehydrogenation of LCO is clearly evident with loadings of 30 wt% Fe on activated carbon support, exhibiting the greatest hydrogen yield. Interestingly, at longer catalytic run-times we see the product transition shifting through a reduction in hydrogen produced to an increasing amount of industrially important light olefins.

## 6. The microwave-initiated catalytic deconstruction of plastics—waste to hydrogen and carbon nanomaterials

**Figure 12.**
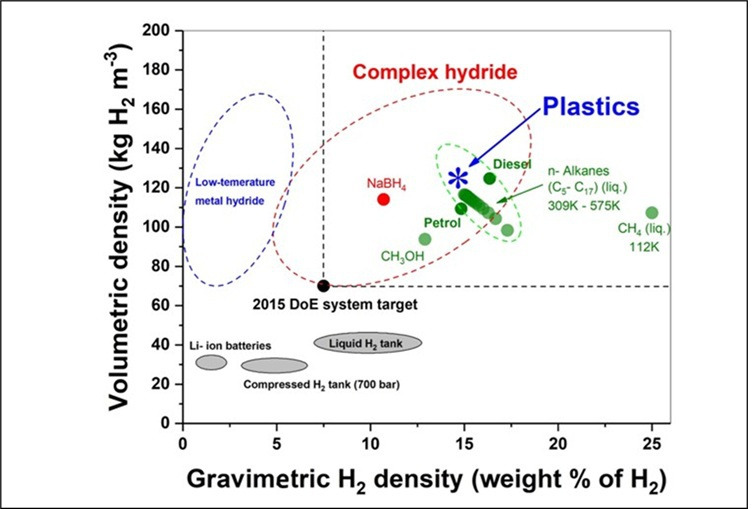
Volumetric and gravimetric hydrogen densities for a range of hydrogen storage materials. Note the high hydrogen densities for typical plastics. The US Department of Energy (DoE) 2015 system target is also shown. The plastics shown in the figure represent thermoplastics composed solely of hydrogen (H) and carbon (C), such as polyethylene, polypropylene and polystyrene [23].

The basic philosophy here was to extend our microwave-initiated catalysis approach to the deconstruction or decomposition; our view was that plastics waste should be seen (well, *must* be seen) as highly effective, anthropogenically produced hydrogen storage materials and as a source, not only of hydrogen, but also carbon. For example, the world’s most widely used and produced plastic, polyethylene, contains some 14 weight% hydrogen and 86% carbon (figure 12).

**Figure 13.**
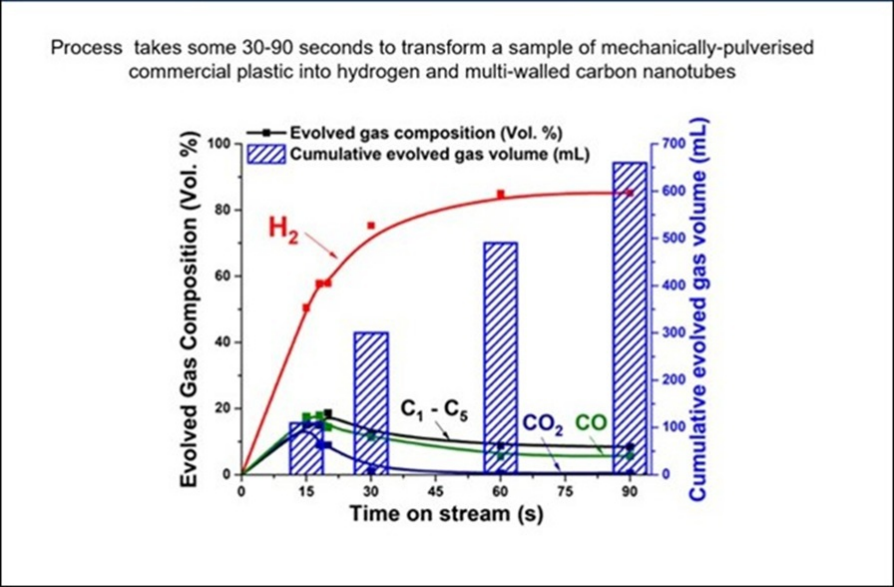
Microwave-initiated hydrogen stripping from polyethylene illustrating the time dependence of hydrogen evolution. Also shown are the time-dependent levels of carbon species C1–C5 as well as the oxides of carbon, CO and CO_2_ [23].

Microwave-driven catalysis proves to be an exceptionally fast and efficient route for hydrogen stripping from plastics. The process almost instantly starts, with hydrogen levels surging to approximately 80 vol% within just 30 s and complete decomposition of HDPE occurring in only 20 s (figure 13). Over a total reaction time of around 90 s, the plastic feedstock is dehydrogenated, leaving behind solid carbon, in this case high purity multi-walled carbon nanotubes.

**Figure 14.**
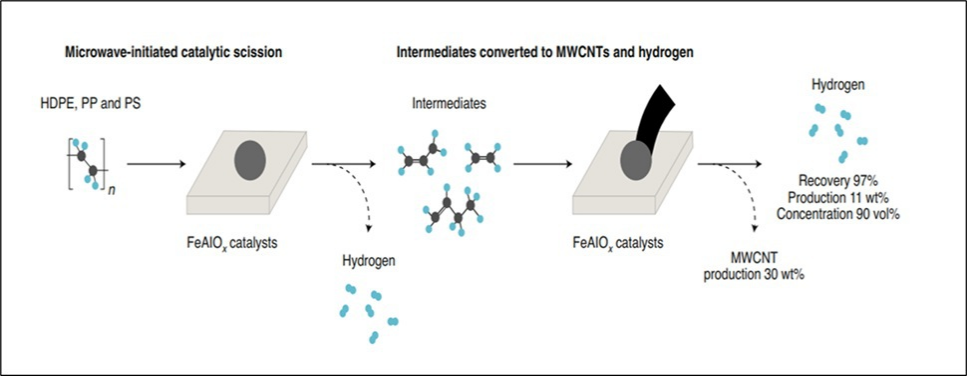
A schematic representation of a proposed mechanism for the microwave-initiated catalytic deconstruction of model plastics HDPE, PP and PS to hydrogen and carbon nanotubes. (Reproduced from [52], with permission from Springer Nature.)

Microwave-initiated catalytic deconstruction of a wide range of plastics back to their elemental constituents, therefore, produces high yields of both hydrogen and carbon, the latter as nanomaterials and in many cases carbon nanotubes. A schematic representation of the proposed mechanism for the deconstruction of plastics to hydrogen and carbon nanotubes is given in figure 14.

The unique interaction between catalyst particles and plastic polymers under microwave initiation plays a crucial role in achieving high efficiency and selectivity for hydrogen and carbon nanotube production. Unlike the Fe catalysts, plastics are largely transparent to microwave energy and remain cold during irradiation. By contrast, catalyst particles act both as energy converters and as active sites for the reaction, owing to their electrodynamic properties and charge dynamics, which influence their susceptibility to microwave energy. This difference in material properties directly impacts the heating rate, reaction pathways and catalytic mechanisms.

In this process, heat is generated locally at the catalyst and then transferred to the plastic reactants. This selective heating creates a distinct thermal flux, with heat flowing outward from the catalyst surface, aligning with the direction of mass transfer. This promotes the desorption of neutral hydrogen (presumably in molecular form) from active sites on the catalyst. This mechanism contrasts sharply with conventional heating, where catalytic reactions are often limited by slower molecular desorption rates due to uniform bulk heating.

This selective heating effect is critically important for minimizing undesired side reactions, such as thermal decomposition of plastic polymers, which typically produce a complex mixture of hundreds or even thousands of by-products. Instead, we propose that the primary catalytic mechanism in this system involves the microwave-initiated cleavage of C–H bonds, offering a highly targeted and efficient approach to plastic conversion.

## 7. Summary

We hope to have illustrated that microwave-initiated catalytic decomposition or deconstruction of fossil hydrocarbons and plastics waste can yield large amounts of hydrogen—in some cases with near-zero or zero CO_2_ emissions in the production process as well as valuable carbon co-products. Advantages of this microwave-initiated catalysis approach derive from unique benefits such as selective heating, fast response time and the minimization of secondary reactions. The fundamental approach is that the catalyst particles themselves—if they are in the correct mesoscopic size regime close to the Size-Induced Metal-Insulator Transition—are highly capable of absorbing microwaves, transforming the incoming electromagnetic radiation into heat to then initiate the rapid and high catalytic activity, particularly in this critical particle size-regime. Microwave-initiated catalysis achieves the desired reaction temperature with ease as the C–H bonds on the surface of the Fe catalysts results in the production of hydrogen and high-value nanoscale carbonaceous materials (figure 14) solid. The resulting hydrogen—in all cases—can clearly quickly dissociate from the active surface sites of the catalyst and this clearly also avoids the many side reactions that one encounters in the conventional thermal activation of heterogeneous catalytic reactions.

In the case of the simplest saturated hydrocarbon, methane, microwave-initiated catalysis leading to hydrogen and solid nanoscale carbon product is particularly attractive since it will allow the use of available infrastructure for the delivery of natural gas *directly* to the conversion point to hydrogen and solid carbon. Of course, such a catalytic decomposition of methane would yield large amounts of the carbon co-product (some 3 tons of carbon for every ton of H_2_ from reaction (1.3)). However, we note there is a growing market for the carbon by-products, most notable (and attractive) high-value carbon markets would be Li-ion battery-grade graphitic carbon. The latter is therefore a highly attractive co-product for rapid development. This approach therefore not only inputs to the pursuit of the hydrogen economy but also in battery electric vehicles.

Of course, many challenges remain in scaling-up microwave-initiated pyrolysis of fossil fuels and plastics waste. These include (i) a complete analysis and comparison of (conventional) thermal and microwave heating. Approximate estimates, so far, indicate that microwave heating uses about half the electrical power of conventional heating. This will be critical for determining the precise optimal temperature of the operating catalyst—yet to be determined, as these present studies use only an optical pyrometer to measure the catalyst temperature; (ii) perhaps the greatest challenge is maintaining the functional (operational) integrity of the catalyst as a hydrocarbon is dehydrogenated and carbon is deposited on the catalyst surface, deactivating it from supporting hydrogen production. Burning off carbon on the catalyst surface to regenerate its initial activity would cancel any significant advantages over, say, a conventional S-MR process because of the resulting CO_2_ emissions. Steam treatment would be beneficial as the CO formed (with hydrogen)—essentially producing Syngas—would be very useful; (iii) solid carbon must obviously be continuously removed from the pyrolysis reactor together, for example, with the Fe catalyst that is undoubtedly contaminated with carbon—indeed in many cases forming iron carbides, themselves exhibiting catalytic properties. As noted, a clear target, high-value carbon material is surely high-purity synthetic graphite. And last—but certainly not least—a major challenge is the precise nature of the elementary reactions and the rate-determining steps in the microwave-initiated processes at the catalyst surface.

Over two decades ago, Armor, in two insightful and prescient surveys, noted ‘Catalysis will play multiple roles in all aspects of H_2_ production’. We believe that these clear, multiple needs will be further satisfied by the movement from conventional, thermal-initiated catalysis to microwave-initiated catalysis—a new, burgeoning and exciting area of heterogeneous catalysis. These advances can open the possibility of using fossil fuel hydrocarbons for the production of sustainable hydrogen and high-value carbon nanomaterials, particularly synthetic graphite. Overall, this approach amounts to the decarbonization of fossil fuels.

## Data Availability

This article has no additional data.

## References

[1] Alvera M. 2021 The hydrogen revolution. London, UK: Hodder & Stoughton Ltd.

[2] Hydrogen Program. 2023 US national clean energy strategy and roadmap. Washington, DC: United States Department of Energy Press.

[3] U.S. Department of energy. 2020 Hydrogen strategy enabling a low-carbon economy. Washington, DC: Office of Fossil Energy, United States Department of Energy Press.

[4] Abe JO *et al*. 2019 Hydrogen energy, economy and storage: review and recommendation. Int. J. Hydrog. Energy **44**, 15072–15086. (10.1016/j.ijhydene.2019.04.068)

[5] Bicelli P. 1986 Hydrogen: a clean energy source. Int. J. Hydrog. Energy **11**, 555–562. (10.1016/0360-3199(86)90121-7)

[6] Policy@Manchester. On hydrogen. pp. 3–33. Manchester, UK: University of Manchester. See https://www.policy.manchester.ac.uk/publications/on-hydrogen/.

[7] Martin P *et al*. 2024 A review of challenges with using the natural gas system for hydrogen. Energy Sci. Eng **12**, 3995–4009. (10.1002/ese3.1861)

[8] Armor JN. 1999 The multiple roles for catalysis in the production of H2. Applied Catalysis A **176**, 159–176. (10.1016/S0926-860X(98)00244-0)

[9] Armor JN. 2005 Catalysis and the hydrogen economy. Catal. Lett **101**, 131–135. (10.1007/s10562-005-4877-3)

[10] Song C, Gaffney A, Fujimoto K. 2002 CO₂ conversion and utilization. Washington, DC: American Chemical Society, Division of Petroleum Chemistry.(ACS Symposium Series 809). (10.1021/bk-2002-0809)

[11] Herzog H. 2022 Direct air capture. In Greenhouse gas removal technologies, pp. 115–137. Cambridge, UK: The Royal Society of Chemistry. (10.1039/9781839165245-00115)

[12] Herzog H. 2024 Getting real about capturing carbon from the air. One Earth **7**, 1477–1480. (10.1016/j.oneear.2024.08.011)

[13] Van Noorden R. 2010 Carbon sequestration: buried trouble. Nature New Biol **463**, 871–873. (10.1038/463871a) 20164897

[14] Steinberg S, Cheng HS. 1989 Modern and prospective technologies for hydrogen production from fossil fuels. Int. J. Hydrog. Energy **14**, 797–820. (10.1016/0360-3199(89)90018-9)

[15] Muradov NZ. 1993 How to produce hydrogen from fossil fuels without CO2 emission. Int. J. Hydrog. Energy **18**, 211–215. (10.1016/0360-3199(93)90021-2)

[16] Muradov NZ. 1998 CO2-free production of hydrogen by catalytic pyrolysis of hydrocarbon fuel. Energy Fuels **12**, 41–48. (10.1021/ef9701145)

[17] Steinberg S. 1999 Fossil fuel decarbonization technology for mitigating global warming. Int. J. Hydrog. Energy **24**, 771–777. (10.1016/S0360-3199(98)00128-1)

[18] Muradov NZ. 2017 Low to near-zero CO2 production of hydrogen from fossil fuels: status and perspectives. Int. J. Hydrog. Energy **42**, 14058–14088. (10.1016/j.ijhydene.2017.04.101)

[19] Jie X *et al*. 2019 The decarbonisation of petroleum and other fossil hydrocarbon fuels for the facile production and safe storage of hydrogen. Energy Environ. Sci **12**, 238–249. (10.1039/C8EE02444H)

[20] Gonzales-Cortes S *et al*. 2016 Wax: a benign hydrogen-storage material that rapidly releases H₂-rich gases through microwave-assisted catalytic decomposition. Sci. Rep **6**, 1–11. (10.1038/srep35315) 27759014 PMC5069496

[21] Jie X *et al*. 2017 Rapid production of high‐purity hydrogen fuel through microwave‐promoted deep catalytic dehydrogenation of liquid alkanes with abundant metals. Angew. Chem. Int. Ed **56**, 10170–10173. (10.1002/anie.201703489) 28544164

[22] Yao B *et al*. 2018 H₂–rich gas production from leaves. Catal. Today **317**, 43–49. (10.1002/anie.201703489)

[23] Jie X *et al*. 2020 Microwave-initiated catalytic deconstruction of plastic waste into hydrogen and high-value carbons. Nat. Catal **3**, 902–912. (10.1038/s41929-020-00518-5)

[24] Jie X *et al*. 2022 Size-dependent microwave heating and catalytic activity of fine iron particles in the deep dehydrogenation of hexadecane. Chem. Mater **34**, 4682–4693. (10.1021/acs.chemmater.2c00630) 35645460 PMC9134345

[25] Perenboom JAAJ, Wyder P, Meier F. 1981 Electronic properties of small metallic particles. Phys. Rep **78**, 173–292. (10.1016/0370-1573(81)90194-0)

[26] Halperin WP. 1986 Quantum size effects in metal particles. Rev. Mod. Phys. **58**, 533–606. (10.1103/RevModPhys.58.533)

[27] Frohlich H. 1937 Die spezifische wärme der electronen kleiner metallteilchen bei tiefen temperaturen. Physica (Utr) **4**, 406–412.

[28] Kubo R. 1962 Electronic properties of metallic fine particles. J. Phys. Soc. Jap **17**, 975–986. (10.1143/JPSJ.17.975)

[29] Edwards PP, Sienko MJ. 1978 Universality aspects of the metal-nonmetal transition in condensed media. Phys. Rev. B **17**, 2575–2581. (10.1103/PhysRevB.17.2575)

[30] Edwards PP, Sienko MJ. 1982 The transition to the metallic state. Acc. Chem. Res **15**, 87–93. (10.1021/ar00075a004)

[31] Edwards PP, Sienko MJ. 1983 What is a metal? Int. Revs. in Phys Chem **3**, 83–137. (10.1080/01442358309353340)

[32] Edwards PP, Rao CNR. 1985 The metallic and non-metallic states of matter. London, UK: Taylor and Francis Ltd.

[33] Edwards PP. 1990 The metallic state – revisited. In Advances in physical metallurgy (eds JA Charles, GC Smith). London, UK: The Institute of Metals.

[34] Edwards PP, Ramakrishnan TVR, Rao CNR. 1995 The metal-nonmetal transition: a global perspective. J. Phys. Chem **99**, 5228–5239. (10.1021/J100015A002)

[35] Edwards PP, Johnston RL, Rao CNR, Tunstall D, Hensel F. 1998 The metal–insulator transition: a perspective. Phil. Trans. R. Soc. A **356**, 5–22. (10.1098/rsta.1998.0146)

[36] Edwards PP, Johnson RL, Hensel F, Rao C, Tunstall DP. 1999 A perspective on the metal-nonmetal transition. Solid State Phys **52**, 229–338. (10.1016/S0081-1947(08)60020-X)

[37] Edwards PP, Lodge MTJ, Hensel F, Redmer R. 2010 … a metal conducts and a non-metal doesn’t. Phil. Trans. R. Soc. A **368**, 941–965. (10.1098/rsta.2009.0282) 20123742 PMC3263814

[38] Hensel F, Slocombe DR, Edwards PP. 2015 On the occurrence of metallic character in the periodic table of the chemical elements. Phil. Trans. R. Soc. A **373**, 20140477. (10.1098/rsta.2014.0477) 25666074

[39] Edwards PP. 1986 Divided metals. Proc. Indian Natl. Sci. Acad **A52**, 265–291.

[40] Nimtz G, Marquardt P. 1988 Size-induced metal-insulator transition in metals and semiconductors. J. Cryst. Growth **86**, 66–71. (10.1016/0022-0248(90)90700-U)

[41] Edwards PP, Johnston RL, Rao CNR. 1999 On the size‐induced metal‐insulator transition in clusters and small particles. In Metal clusters in chemistry (eds P Braunstein, LAP Oro, PR Raithby), pp. 1454–1481. Weinheim, Germany: Wiley-VCH Verlag Gmbh. (10.1002/9783527618316)

[42] Rao CNR *et al*. 2000 Metal nanoparticles and their assemblies. Chem. Soc. Rev **29**, 27–35. (10.1039/A904518J)

[43] Edwards PP, Thomas JM. 2007 Gold in a metallic divided state—from Faraday to present‐day nanoscience. Angew. Chem. Int. Ed **46**, 5480–5486. (10.1002/anie.200700428) 17562538

[44] DiCenzo S, Wertheim G. 1985 Clusters of atoms and molecules II, (ed. H Haberland), pp. 361–383. Berlin, Germany: Springer-Verlag.

[45] Faraday M. 1857 The Bakerian lecture: experimental relations of gold (and other metals) to light. Phil. Trans. R. Soc. B **147**, 145–181. (10.1098/rstl.1857.0011)

[46] Marquardt P, Nimtz G, Heite G, Peters H. 1988 Microwave evidence for a size-induced metal-insulator transition in mesoscopic conductors. Mat. Res. Soc. Symp. Proc **124**, 155–160. (10.1557/PROC-124-155)

[47] Marquart P, Nimtz G. 1989 Fest korper probleme, pp. 317–328, vol. **29**. Oxford, UK: Pergamon.

[48] Marquart P, Nimtz G. 1991 Size-dependent dielectric response of small metal particles. Phys. Rev. B **43**, 14245–14247. (10.1103/PhysRevB.43.14245) 9997298

[49] Schaefer FC, v. Baltz R. 1995 Conductivity of a small metallic particle. Ann. Der Phys **4**, 191–201. (10.1002/ANDP.19955070305)

[50] Porch A, Slocombe DR, Edwards PP. 2013 Microwave absorption in powders of small conducting particles for heating applications. Phys. Chem. Chem. Phys **15**, 2757–2763. (10.1039/C2CP43310A) 23321957

[51] Aldrees SA. 2022 Hydrogen production from crude oil using microwave-initiated catalytic technology. D. Phil Thesis, Oxford University.

[52] Lopez G, Santamaria L. 2020 Microwaving plastic into hydrogen and carbons. Nat. Catal **3**, 861–862. (10.1038/s41929-020-00538-1)

